# Patients hospitalized with acute heart failure, worsening renal function, and persistent congestion are at high risk for adverse outcomes despite current medical therapy

**DOI:** 10.1002/clc.24080

**Published:** 2023-07-18

**Authors:** Lauren Cooper, Adam DeVore, Jennifer Cowger, Sean Pinney, David Baran, Tracy A. DeWald, Tara Burt, Jan B. Pietzsch, Antony Walton, Keith Aaronson, Palak Shah

**Affiliations:** ^1^ Department of Cardiology North Shore University Hospital Manhasset New York USA; ^2^ Inova Heart & Vascular Institute Inova Fairfax Hospital Falls Church Virginia USA; ^3^ Department of Medicine Duke University School of Medicine Durham North Carolina USA; ^4^ Division of Cardiovascular Medicine Henry Ford Hospitals Detroit Michigan USA; ^5^ Heart & Vascular Center University of Chicago Medicine Chicago Illinois USA; ^6^ Cleveland Clinic Heart Weston Florida USA; ^7^ Procyrion Houston Texas USA; ^8^ Wing Tech Inc Menlo Park California USA; ^9^ Alfred Health Melbourne Victoria Australia; ^10^ Department of Internal Medicine University of Michigan Ann Arbor Michigan USA

**Keywords:** heart failure, heart failure with preserved ejection fraction, heart failure with reduced ejection fraction, worsening renal failure

## Abstract

**Introduction:**

Approximately 1/3 of patients with acute decompensated heart failure (ADHF) are discharged with persistent congestion. Worsening renal function (WRF) occurs in approximately 50% of patients hospitalized for ADHF and the combination of WRF and persistent congestion are associated with higher risk of mortality and HF readmissions.

**Methods:**

We designed a multicenter, prospective registry to describe current treatments and outcomes for patients hospitalized with ADHF complicated by WRF (defined as a creatinine increase ≥0.3 mg/dL) and persistent congestion at 96 h. Study participants were followed during the hospitalization and through 90‐day post‐discharge. Hospitalization costs were analyzed in an economic substudy.

**Results:**

We enrolled 237 patients hospitalized with ADHF, who also had WRF and persistent congestion. Among these, the average age was 66 ± 13 years and 61% had a left ventricular ejection fraction (LVEF) ≤ 40%. Mean baseline creatinine was 1.7 ± 0.7 mg/dL. Patients with persistent congestion had a high burden of clinical events during the index hospitalization (7.6% intensive care unit transfer, 2.1% intubation, 1.7% left ventricular assist device implantation, and 0.8% dialysis). At 90‐day follow‐up, 33% of patients were readmitted for ADHF or died. Outcomes and costs were similar between patients with reduced and preserved LVEF.

**Conclusions:**

Many patients admitted with ADHF have WRF and persistent congestion despite diuresis and are at high risk for adverse events during hospitalization and early follow‐up. Novel treatment strategies are urgently needed for this high‐risk population.

## Graphical abstract

Acute heart failure with worsening renal function and persistent congestion is common and represents a high‐risk and for which there are inadequate treatment options. Novel treatment strategies are urgently needed for this high‐risk population.

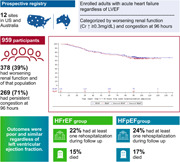

**Abbreviations**
ADHF: acute decompensated heart failure
CRS: cardiorenal syndrome
CRT: cardiac resynchronization therapy
HF: heart failure
HFpEF: heart failure and preserved ejection fraction
HFrEF: heart failure and reduced ejection fraction
ICD: implantable cardioverter defibrillator
ICU: intensive care unit
LVAD: left ventricular assist device
PC: persistent congestion
WRF: worsening renal function

## 1 INTRODUCTION

Heart failure (HF) is common, affecting more than six million adults and resulting in over 1.8 million hospitalizations per year in the United States alone, with significant financial impact.^1^ HF is a chronic, progressive condition, marked by episodes of acute decompensation, typically requiring hospitalization for stabilization and improvement in symptoms. Almost 90% of hospitalizations are due to symptoms related to volume overload. Unfortunately, outcomes after hospitalization for acute decompensated heart failure (ADHF) are poor. The 1‐year mortality rate after an HF hospitalization has remained high at 20%–30%, and there is additive risk with each subsequent hospitalization.^2^, ^3^, ^4^ For patients with ADHF, many medical therapies have been tested in this patient population without success^5^, ^6^, ^7^ and, as a result, ADHF care remains largely homogenous and unchanged over the past 40 years, with treatment largely focusing on diuresis with intravenous diuretics.^8^, ^9^, ^10^ With currently available treatment options, approximately 33% of patients hospitalized for ADHF are discharged with persistent congestion,^11^, ^12^ and higher degrees of clinical congestion at hospital discharge are associated with increased HF readmissions and reduced survival.^13^, ^14^, ^15^

Moderate to severe renal dysfunction or worsening renal failure (WRF) complicates approximately 66% of ADHF admissions.^16^ The pathophysiology underlying cardiorenal syndrome (CRS) in ADHF is poorly understood and probably multifactorial, reflecting underlying renal dysfunction from HF and other conditions, neurohormonal activation and inflammation, side effects of acute therapies, and renal congestion.^17^ The degree of acute renal dysfunction has not been shown to correlate with the degree of left ventricular systolic dysfunction in heart failure with reduced ejection fraction (HFrEF), though it may be associated with the severity of diastolic dysfunction in heart failure with preserved ejection fraction (HFpEF).^18^ Patients with WRF represent a particularly high‐risk population with poor outcomes. A previously developed and validated predictive model for mortality from the Acute Decompensated Heart Failure National Registry (ADHERE) database identified laboratory indices of renal function to be closely associated with mortality.^19^ A subsequent analysis from this registry of >110 000 admissions for ADHF determined that in‐hospital mortality increased from 1.9% for patients with normal renal function to 7.6% in patients with severe renal dysfunction.^16^ A separate analysis of >10 000 patients from the Swedish Heart Failure (SwedeHF) and Stockholm Creatinine Measurement (SCREAM) registries further demonstrated that WRF within one year was strongly associated with subsequent long‐term mortality in all left ventricular ejection fraction (LVEF) groups.^20^ Together, these data suggest WRF represents a separate and distinct risk factor in both HFrEF and HFpEF.

There are no current proven treatments for patients with CRS. Therapies that reduce congestion without causing WRF have been elusive. The American College of Cardiology Foundation/American Heart Association (ACCF/AHA) Heart Failure Guidelines previously identified the lack of recognition and treatment of CRS as a key gap in heart failure knowledge and evidence.^19^ There is a need to better understand this unique patient population to develop new treatment strategies that will favorably alter the course of this condition.

## 2 METHODS

### 2.1 Study population

This multicenter, prospective registry study enrolled participants from 12 clinical sites in the United States and Australia. All adult patients aged ≥21 years admitted with a primary diagnosis of ADHF, regardless of LVEF, were screened. Informed consent was obtained from participants who met enrollment criteria and agreed to participate. Participants were categorized into one of three groups, ADHF alone, ADHF + WRF, and ADHF + WRF + persistent congestion at 96‐h post‐admission. Data collection varied by group, with the ADHF + WRF + persistent congestion group having the most extensive data collection. Due to the observational nature of this study, informed consent requirements were based on the local requirements of each participating institution and varied in level. For example, some sites require that all participants provide written informed consent before any screening took place, while others only required participants to provide written informed consent if they were assigned to the full data collection group (ADHF + WRF + persistent congestion). The institutional review boards at each of the 12 study sites approved the protocol and consent forms. Participants were enrolled from April 2019 to March 2020.

An increase in creatinine of ≥0.3 mg/dL from baseline was used to define WRF, consistent with prior studies.^20^, ^21^ The baseline creatinine and WRF were determined by site investigators from data obtained during the hospitalization or in the 90 days before hospital admission. The presence of persistent congestion at 96‐h post‐admission was defined by signs, symptoms, or objective findings of HF. If hemodynamic measurements were obtained, persistent congestion was defined as PCWP > 22 mmHg or CVP > 10 mmHg. In the absence of hemodynamic measurements, physical exam findings (e.g., lower extremity edema, ascites, elevated jugular venous pressure, or pulmonary rales), clinical symptoms (e.g., dyspnea at rest or with minimal exertion, paroxysmal nocturnal dyspnea, or orthopnea), and confirmatory tests (e.g., chest x‐ray or echocardiography) were used.

Participants were excluded if they had acute kidney failure, defined as serum creatinine ≥4.0 mg/dL or stage V chronic kidney disease (eGFR ≤ 15 mL/min/1.73 m^2^) on admission or within the prior 90 days. Participants were excluded if they had a single kidney or required dialysis or ultrafiltration in the prior 90 days. Participants were also excluded if they were supported with a mechanical support device or had a prior heart transplant at the time of enrollment.

Enrolled participants were treated according to the standard of care at each enrolling facility and followed for significant clinical events during the hospitalization and through 90 days post‐discharge (ADHF + WRF + persistent congestion group only). Eight US sites participated in an economic substudy and participants at those sites signed additional consent approving the collection of billing data.

### 2.2 Data source

Data were collected from medical records and follow‐up phone calls to ascertain vital status and outcomes at 90 days. Records were reviewed for details regarding past medical history, the presence of comorbidities, and any history of HF or prior hospitalizations. Exam findings, blood, and urine laboratory results, imaging studies, invasive testing, and treatments were similarly reviewed. For participants participating in the economic data collection, billing data were collected for the index hospitalization and subsequent rehospitalizations within 90 days of discharge from the index hospitalization. Participants who were readmitted within 90 days of discharge had all costs of care analyzed, even if the hospitalization extended past 90 days.

### 2.3 Outcomes

Significant clinical events during the initial hospitalization included escalation of care to an intensive care unit (ICU), mechanical ventilation, extracorporeal membrane oxygenation, dialysis, mechanical circulatory support, discharge on inotropes, addition to the transplant list, or death.

Outcomes at 90 days (±14 days) included survival, HF rehospitalization, and progression of disease, defined as need for evaluation for a left ventricular assist device (LVAD), addition to the heart transplant waitlist, or undergoing a heart transplantation. Costs and length of stay (LOS) were analyzed for subjects participating in the economic substudy.

### 2.4 Statistical analysis

The data are available on request from the authors. Baseline characteristics, symptoms, and significant clinical events were described by count and percentage of study population. All descriptive data were compared using chi‐square test for two‐way tables. In cases when frequencies below 5 were met for more than 20% of the cells, the Fisher exact test was applied. All continuous data were compared using a two‐independent sample *t* test. In cases when data failed the Shapiro–Wilk normality test with *p* value < .15, nonparametric Wilcoxon rank‐sum test was applied.

Univariable analysis was performed to assess the relationship between baseline characteristics and the primary study outcome. Kaplan–Meier curves were generated for event‐free survival by analysis of time (days) from 96‐h post‐admission to the first hospitalization (when persistent congestion despite medical management was confirmed) until the admission to subsequent hospitalization, death from any reasons, LVAD, or heart transplantation, whichever occurred first. This analysis was conducted for all participants within the corresponding subgroup (ADHF + WRF with persistent congestion, HFrEF vs. HFpEF) and censored with the date of follow‐up. In cases when follow‐up information was not collected or survival status was unknown, the time was censored with the date of discharge from the last hospitalization. In cases when data were available >90‐day post‐discharge, the time was censored at 90‐day post‐discharge. Participants with no follow‐up were not included (*n* = 32).

Hospitalization costs were calculated by multiplying all charges by the cost center‐specific cost‐to‐charge ratio obtained from each hospital's Medicare cost report. Physician fees were not analyzed. LOS for the economic substudy participants was collected from medical records. All resource utilization and cost data were analyzed by institution, and in aggregate. Continuous variables were described as mean ± SD along with median (interquartile range [IQR]), as appropriate. Univariate comparisons between sites and between subcohorts with specific characteristics were performed using independent *t* tests. All analyses were conducted using JMP 15 (SAS Institute).

## 3 RESULTS

### 3.1 Baseline characteristics

A total of 1210 participants were enrolled in this study of which there were 41 screen failures and 210 participants who could not be classified. Out of the remaining 959 participants, 378 (39%) had WRF and of that population 269 (71%) had persistent congestion. Of the 269 classified to ADHF + WRF + persistent congestion, 237 provided consent to participate in the full data collection (Supporting Information: Figure).

Of the participants admitted with ADHF who also had WRF and persistent congestion at 96 h, the average age was 66 (±13) years, 66% were male, and 23% were Black participants (Table 1). Chronic kidney disease was present in 39% of participants, diabetes mellitus in 47%, history of atrial fibrillation in 50%, and a history of hypertension in 68%. At admission, 148 participants (62%) had NYHA Class III or IV symptoms. There were 144 (61%) participants with EF ≤ 40%, 14 (5.9%) with EF 41%–49%, and 75 (32%) with EF 50%–75%.

**Table 1:** Baseline patient characteristics.

Characteristic	ADHF + WRF with persistent congestion (*N* = 237)	HFrEF (*N* = 144)	HFpEF (*N* = 89)	*p* Value (HFrEF vs. HFpEF)^a^
Sex, *n* (%)				.046
Male	156 (65.8)	101 (70.1)	51 (57.3)	
Female	81 (34.2)	43 (29.9)	38 (42.7)	
Age (years), mean	66	64.2 (12.87)	68.6 (11.91)	.012
Race, *n* (%)				.416
Aboriginal	2 (0.8)	1 (0.7)	1 (1.1)	
Asian Pacific	2 (0.8)	1 (0.7)	1 (1.1)	
Black/African American	55 (23.2)	37 (25.7)	18 (20.2)	
Native Hawaiian/Other Pacific Islander	2 (0.8)	0	2 (2.2)	
White	168 (70.9)	101 (70.1)	63 (70.8)	
Other	8 (3.4)	4 (2.8)	4 (4.5)	
Ethnicity, *n* (%)				.37
Hispanic/Latino	5 (2.1)	2 (1.4)	3 (3.4)	
Not Hispanic/Latino	232 (97.9)	142 (98.6)	86 (96.6)	
LVEF (%) by categories, *n* (%)				
≤25%	100 (42.2)			
26%–40%	44 (18.6)			
41%–49%	14 (5.9)			
50%–75%	75 (31.6)			
Unknown	4 (1.7)			
NYHA functional class at admission, *n* (%)				.319
Class I	2 (0.8)	0	2 (2.2)	
Class II	16 (6.8)	11 (7.6)	5 (5.6)	
Class III	73 (30.8)	46 (31.9)	24 (27.0)	
Class IV	75 (31.6)	51 (35.4)	24 (27.0)	
Unknown	71 (30.0)	36 (25.0)	34 (38.2)	
Past medical history, *n* (%)				
Chronic kidney disease	92 (38.8)	57 (39.6)	34 (38.2)	.834
Diabetes mellitus	111 (46.8)	66 (45.8)	44 (49.4)	.592
Hyperlipidemia	56 (23.6)	39 (27.1)	17 (19.1)	.166
Hypertension	160 (67.5)	91 (63.2)	67 (75.3)	.055
Atrial fibrillation	118 (49.8)	64 (44.4)	52 (58.4)	.038
Coronary artery disease	115 (48.5)	72 (50.0)	40 (44.9)	.453
Medications before or started at admission, *n* (%)				.034
Loop diuretics	162 (68.4)	28 (19.4)	12 (13.5)	
Other diuretics	46 (19.4)	95 (66.0)	66 (74.2)	
Beta‐blocker	144 (60.8)	23 (16.0)	23 (25.8)	
ACE inhibitor	40 (16.9)	98 (68.1)	44 (49.4)	
ARB	18 (7.6)	10 (6.9)	8 (9.0)	
ARNI	13 (5.5)	13 (9.0)	0	
MRA	82 (34.6)	54 (37.5)	28 (31.5)	
Inotropes	6 (2.5)	6 (4.2)	0	
Hydralazine and nitrates	41 (17.3)	24 (16.7)	17 (19.1)	
Antiarrhythmics	28 (11.8)	21 (14.6)	7 (7.9)	
Digoxin	25 (10.5)	18 (12.5)	7 (7.9)	
Ivabradine	2 (0.8)	1 (0.7)	1 (1.1)	
Diabetic medication	24 (10.1)	16 (11.1)	8 (9.0)	
Insulin	16 (6.8)	9 (6.3)	7 (7.9)	
SGLT2 inhibitors	1 (0.4)	1 (0.7)	0	
Other	13 (5.5)	10 (6.9)	3 (3.4)	
Vitals and laboratory values on admission, median (Q1, Q3)				
SBP (mmHg), *n* = 233	120 (104, 137)	115 (99, 135)	127 (112, 140)	<.001
Creatinine (mg/dL)	1.60 (1.20, 2.20)	1.6 (1.2, 2.2)	1.6 (1.2, 2.2)	.084
Brain natriuretic peptide (ng/dL), *n* = 139	980 (423, 2425)	*n* = 81 1883 (886, 2840)	*n* = 55 488 (175, 934)	<.001
N‐terminal Pro B‐type natriuretic peptide (ng/L), *n* = 51	4492 (1792, 11 152)	*n* = 32 7105 (3282, 16 757)	*n* = 19 2257 (986, 6029)	.007

Abbreviations: ACE, angiotensin‐converting enzyme; ARB, angiotensin II receptor blocker; ARNI, angiotensin II receptor blocker/neprilysin inhibitor; MRA, mineralocorticoid receptor antagonist; SBP, systolic blood pressure; SGLT2, sodium–glucose cotransporter.

^a^ All descriptive data (sex, ethnicity, race, and LVEF by categories) were compared using chi‐square test for two‐way tables. In cases when frequencies below 5 were met for more than 20% of the cells, Fisher exact test was applied. All continuous data (age (years) and left ventricular ejection fraction [LVEF; %]) were compared using two‐independent sample *t* test. In cases when data failed Shapiro–Wilk normality test with *p* value < .15, nonparametric Wilcoxon rank‐sum test was applied.

Medications present before admission included loop diuretics (65%), beta‐blockers (61%), angiotensin‐converting enzyme inhibitors/angiotensin II receptor blockers (25%), angiotensin II receptor blocker/neprilysin inhibitors (5.5%), and mineralocorticoid receptor antagonist (35%).

Participants were considered to have persistent congestion based on signs or symptoms at 96 h which included lower extremity edema in 78%, elevated JVP in 45%, and dyspnea at rest or with minimal exertion in 25% (Table 2).

**Table 2:** Findings of persistent congestion assessment at 96 h.

Sign or symptom^a^	ADHF + WRF with PC (*N* = 237)^b^
Lower extremity edema	185 (78.1)
Elevated JVP	107 (45.1)
Dyspnea at rest or with minimal exertion	60 (25.3)
Pulmonary rales	41 (17.3)
Pulmonary vascular congestion on CXR	28 (11.8)
Orthopnea	24 (10.1)
Ascites	18 (7.6)
Paroxysmal nocturnal dyspnea	14 (5.9)
PCWP >/= 22 mmHg	13 (5.5)
CVP >/= 10 mmHg	10 (4.2)
Other	31 (13.1)

Abbreviations: ADHF, acute decompensated heart failure; CVP, central venous pressure; CXR, chest x‐ray; JVP, jugular venous pressure; PC, persistent congestion; PCWP, pulmonary capillary wedge pressure; WRF, worsening renal function.

^a^ Patient could have more than one finding of congestion.

^b^ Seventy‐five patients (31.6%) had only one finding of persistent congestion, and 162 patients (68.4%) had two or more.

### 3.2 Clinical events during index hospitalization and 90‐day follow‐up

During the index hospitalization, 7.6% of participants were transferred to the ICU, 3.4% were discharged on inotropes, and 3.4% of participants died (Table 3). HFrEF and HFpEF participants had similar rates of events during the index hospitalization, except only HFrEF participants were discharged on inotropes (5.6% of HFrEF participants, 0% of HFpEF participants; Supporting Information: Table).

**Table 3:** Significant clinical events during initial ADHF hospitalization.

Clinical event^a^	ADHF + WRF with PC (*N* = 237)
Transfer to ICU, *n* (%)	18 (7.6)
Intubation/mechanical ventilation	5 (2.1)
Balloon pump	1 (0.4)
ECMO	0
LVAD	4 (1.7)
Other MCS device	1 (0.4)
Dialysis	2 (0.8)
Added to transplant list	2 (0.8)
Discharged on Inotropes	8 (3.4)
Death	8 (3.4)
Other^b^	41 (17.3)

Abbreviations: ADHF, acute decompensated heart failure; ECMO, extracorporeal membrane oxygenation; ICU, intensive care unit; LVAD, left ventricular assist device; MCS, mechanical circulatory support; PC, persistent congestion; WRF, worsening renal function.

^a^ Patient could have more than one significant clinical event. If the patient had more than one significant clinical event in one category, the patient is counted only once per category.

^b^ Consisted of, but not limited to, anemia, arrhythmia, catheterization/cardiac surgery, and hypotension.

Overall, serum creatinine increased during admission and decreased back to baseline before discharge. The mean creatinine at baseline was 1.7 ± 0.7 mg/dL, 2.2 ± 0.9 mg/dL at peak, and 1.8 ± 0.8 mg/dL closest to discharge, though this was different for participants with HFrEF compared to HFpEF as described below. Overall, 53% of participants were discharged with clinical signs of persistent congestion. During the 90‐day follow‐up period, 53 participants (23%) had at least one rehospitalization and 29 participants (12%) died.

**Figure 1:**
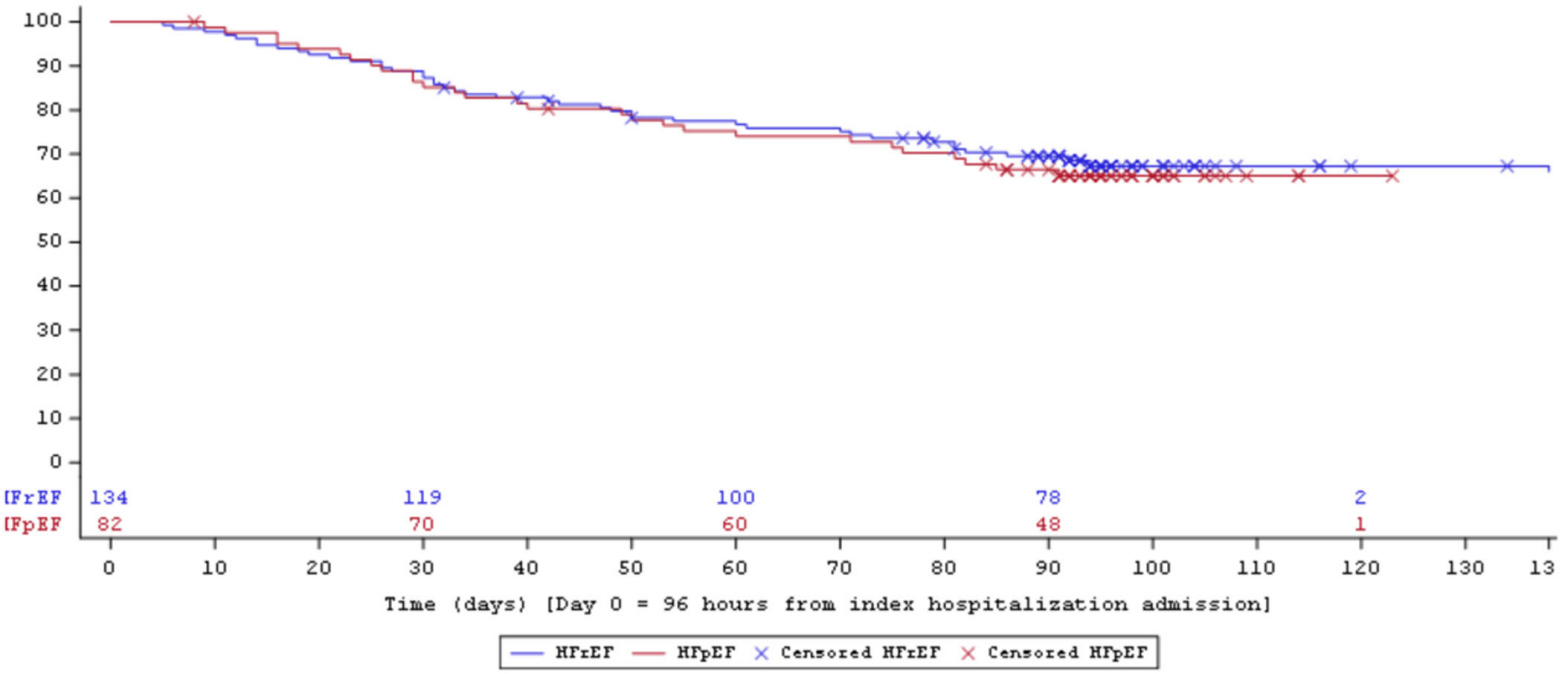
Event‐free survival after hospitalization until rehospitalization, left ventricular assist device implantation, heart transplantation, or death stratified by participants with heart failure with reduced ejection fraction (blue) and heart failure with preserved ejection fraction (red). HFpEF, heart failure with preserved ejection fraction; HFrEF, heart failure with reduced ejection fraction.

At 90‐day follow‐up, 33% of participants with ADHF + WRF + persistent congestion were readmitted for ADHF or died, and 41% were readmitted for ADHF, had an LVAD placed, underwent heart transplant, or died. Event‐free survival until death, LVAD, heart transplantation, or rehospitalization was similar between participants with HFrEF compared to HFpEF (Figure 1).

### 3.3 ADHF in HFrEF versus HFpEF comparison

Of the 233 participants with a documented LVEF, 144 had HFrEF and 89 had HFpEF. The mean age for HFrEF participants was 64 ± 13 years and for HFpEF was 69 ± 12 years (Table 1). For HFrEF participants, 70% were male and 30% were female, whereas for HFpEF participants, 57% were male and 43% were female. Median creatinine at admission was the same for both groups (1.6 mg/dL), but HFrEF participants had a median creatinine of 1.6 mg/dL before discharge whereas participants with HFpEF had an increase to 1.8 mg/dL. Systolic blood pressure on admission was lower in participants with HFrEF (median 115 mmHg vs. 127 mmHg) but median BNP and NT‐proBNP values were higher in participants with HFrEF compared to HFpEF. Events during index hospitalization were similar between the groups. In the HFrEF group, 22% had at least one rehospitalization during follow‐up and 22 participants (15%) died. In the HFpEF group, 24% had at least one hospitalization and 15 participants (17%) died.

### 3.4 Analysis of resource utilization and costs

**Figure 2:**
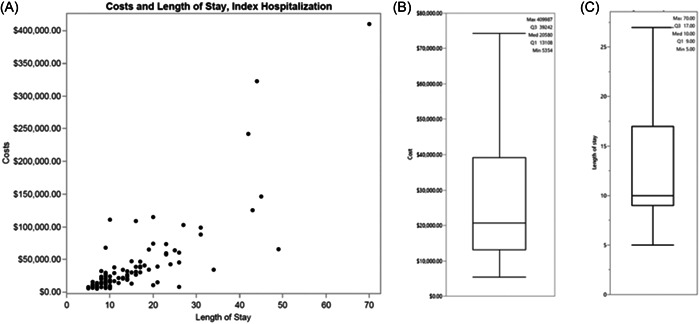
(A) The relationship between cost and length of stay (LOS) for the index hospitalization for acute decompensated heart failure. (B) Boxplot of index hospitalization cost. (C) Boxplot of LOS.

For the index hospitalization, mean costs were $39 343 ± 57 926 and the LOS for participants in the economic substudy was 14.8 ± 10.7 days. Costs and LOS ranged from a minimum of $5435 to a maximum of $409 987, and 5.0–70.0 days, respectively (Figure 2). There was no statistically significant difference in costs between sites for the index ADHF admission. For the first and second rehospitalization, costs were $43 766 ± 69 152 and $20 264 ± 23 444, respectively. Costs were similar between participants with HFrEF and HFpEF (*p* = .09) and were not statistically different (*p* > .05) between participating sites.

Several charges could be identified as outliers. Specifically, four participants received an LVAD during the index admission, and one participant received a heart transplant. Excluding these subjects from the analysis reduced the costs to $30 935 ± 29 361 and LOS to 14.0 ± 9 days.

## 4 DISCUSSION

In this study of patients with ADHF, a large proportion, 39%, developed WRF and of that population, a majority, 71%, also had persistent congestion after 96 h of standard therapy. These participants with ADHF, WRF, and persistent congestion had a high clinical event rate that includes both in‐hospital and short‐term mortality at 90 days, readmission to the hospital for recurrent ADHF, and disease progression leading to LVAD or heart transplantation. When analyzed in this prospective cohort, the cumulative incidence of these events occurred in 42% of our cohort. This study adds to prior findings that patients with HF and renal dysfunction are at a high risk for poor outcomes.^21^ Further, this study is unique in the inclusion and comparison of patients with HFrEF and HFpEF. Despite different baseline demographics, vitals, and laboratory values, patients with HFrEF and HFpEF had similar outcomes. This study also adds to prior work by including an economic analysis showing that the cost for hospitalization for these high‐risk patients is substantial, and that the cost of care remains high even after the first hospitalization.

Most patients admitted with ADHF have volume overload on admission.^22^ The presence of congestion is associated with adverse outcomes^23^, ^24^, ^25^, ^26^, ^27^, ^28^, ^29^, ^30^; and thus a goal of therapy for patients with ADHF is safe and effective decongestion.^23^, ^31^ Moreover, filling pressures have been shown to be more prognostically important than even cardiac index in the management of patients with ADHF, and congestion is associated with worse outcomes independent of cardiac index.^32^ Current recommendations for the treatment of ADHF emphasize the need to assess the trajectory of treatment throughout the hospital course to ensure the patient is progressing toward the goal for discharge.^33^ Despite these efforts, a substantial portion of patients hospitalized with volume overload are discharged with persistent congestion, and persistent congestion is associated with worse outcomes.^14^, ^15^, ^32^, ^33^ In prior studies, the percentage of patients discharged with congestion varies from <10% to almost 50% in part due to the variable ways of measuring and defining congestion. In the current study, 53% of patients with WRF and persistent congestion at 96 h were discharged with remaining clinical signs of congestion, emphasizing that persistent congestion is a high‐risk feature and highlighting the need for effective treatment options for decongestion.

Previous studies have shown that WRF occurs in approximately 1/3 of patients admitted to the hospital for ADHF; the present study observed a similar percentage with 39% of patients having WRF. Those patients with HF and renal dysfunction are at the highest risk for poor outcomes including in‐hospital death and those with renal dysfunction are at high risk of not achieving adequate diuresis.^20^, ^21^ In ADHF patients with WRF, outcomes are poor due to persistent congestion, increased dose of diuretics, and decreased diuretic efficiency.^34^, ^35^, ^36^ Although earlier studies suggested that the risk of readmission for HF and mortality in ADHF was largely mediated by the presence of WRF, follow‐up analyses suggested that the effect of WRF was mediated by the presence of persistent congestion.^20^, ^21^ The combination of WRF and persistent congestion is associated with a greater increase in the risk of mortality and hospital readmission.^20^ In one study, a 30% reduction in eGFR was associated with a 20% increase risk of death; however, the risk of death was modulated by decongestion status, with the risk of death decreasing with decreasing severity of congestion.^37^

Despite the advances in the treatment of chronic heart failure, particularly in HFrEF with many approved and effective therapies for chronic HF,^38^ there have been few advances in the treatment of ADHF, with neutral findings in studies of novel drugs and device strategies for ADHF.^5^, ^6^, ^7^ There is an unmet need for in‐hospital therapies for the treatment of ADHF to augment diuresis and achieve adequate decongestion, including in patients without cardiogenic shock. Therapeutic options may be new medical treatments, such as new diuretic strategies,^39^ or novel devices, such as the Aortix (Procyrion) or preCARDIA (Abbott) devices, intended to improve decongestion.^40^, ^41^

Hospitalization costs of ADHF patients, as demonstrated by the findings of the economic substudy, are substantial, with half of all patients requiring 10 or more days of inpatient care. While these findings are directionally in line with prior studies that reported costs per HF hospitalization in the range of $15 000–$20 000,^42^, ^43^ our data suggest care for ADHF patients with WRF might be somewhat more resource‐intensive and costly than hospitalizations of less complex HF populations. Among other factors, this is evidenced by the study observed LOS of 10.0 days, which is more than twice as high as the reported stay of mean 5.1 days in Medicare's diagnosis‐related group 291, which captures HF patients with major comorbidities and complications. This also results in payments that will likely not cover the costs of care for the patients investigated in the current study.

### 4.1 Limitations

This study has several limitations. There were only 12 sites in the United States and Australia and thus the results may not be generalizable to other regions. In addition, all the sites had dedicated advanced HF programs and their results may be better than in the general clinical community. Though the intention of the study was to enroll consecutively, this was not possible due to local site‐specific informed consent requirements. Data analyzed were limited to the data collected as part of the study, and we relied on accuracy of data entry by study coordinators. Furthermore, associations with the outcome may be influenced by residual measured and unmeasured confounders. Our economic data considered only costs for hospital care, but not for associated physician fees. These might amount to an increase in ~10%–20% additional cost per case.

## 5 CONCLUSIONS

In conclusion, ADHF with WRF and persistent congestion is common among patients hospitalized with ADHF and represents a high‐risk and high‐cost population for which there are inadequate treatment options. These HFrEF and HFpEF patients have a high in‐hospital and 90‐day follow‐up clinical event rate. The findings from this study add to our understanding of the clinical progression of HF and demonstrate the unmet clinical need for novel treatment strategies in CRS.

## Supporting information

Supplemental Figure: Study flow diagram of all participants with acute decompensated heart failure. ADHF: Acute Decompensated Heart Failure; ICF: Informed Consent Form; PC: Persistent Congestion; WRF: Worsening Renal Function.

Supporting information.

## Data Availability

Data available on request from the authors.
