# Supplementary figures and images for: Patients hospitalized with acute heart failure, worsening renal function, and persistent congestion are at high risk for adverse outcomes despite current medical therapy
Source: Clin Cardiol. 2023 Jul 18;46(10):1163–72. doi: 10.1002/clc.24080 (PMC10577559; doi:10.1002/clc.24080)

## Supplemental Figure

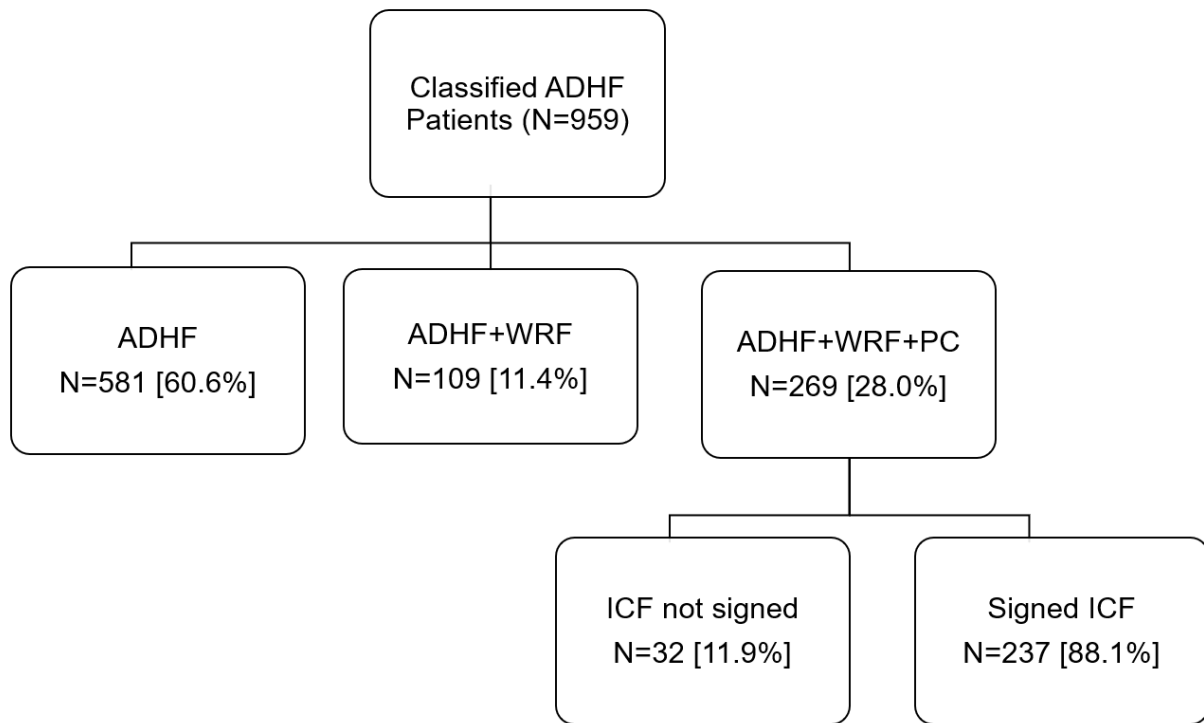

Supplement: Supplementary file 1 — Supplemental Figure: Study flow diagram of all participants with acute decompensated heart failure. ADHF: Acute Decompensated Heart Failure; ICF: Informed Consent Form; PC: Persistent Congestion; WRF: Worsening Renal Function. [file CLC-46-1163-s001.pdf]
