# Supplementary material for: Patients hospitalized with acute heart failure, worsening renal function, and persistent congestion are at high risk for adverse outcomes despite current medical therapy
Source: Clin Cardiol. 2023 Jul 18;46(10):1163–72. doi: 10.1002/clc.24080 (PMC10577559; doi:10.1002/clc.24080)
Supplement: Supplementary file 2 — Supporting information. [file CLC-46-1163-s002.docx]

**Supplemental Table. Significant Clinical Events during Initial ADHF Hospitalization Stratified by HF Type**

| Clinical Event* | HFrEF  (N=144) | HFpEF  (N=89) | Unknown LVEF  (N=4) |
| --- | --- | --- | --- |
| Transfer to ICU, n (%) | 12 (8.3) | 5 (5.6) | 1 (25) |
| Intubation/mechanical ventilation | 3 (2.1) | 2 (2.2) | 0 |
| Balloon Pump | 1 (0.7) | 0 | 0 |
| ECMO | 0 | 0 | 0 |
| LVAD | 4 (2.8) | 0 | 0 |
| Other MCS device | 1 (0.7) | 0 | 0 |
| Dialysis | 1 (0.7) | 1 (1.1) | 0 |
| Added to Transplant List | 2 (1.4) | 0 | 0 |
| Discharged on Inotropes | 8 (5.6) | 0 | 0 |
| Death | 4 (2.8) | 4 (4.5) | 0 |
| Other** | 26 (18.1) | 14 (15.7) | 1(25) |
| *Patient could have more than one significant clinical event. If patient had more than one significant clinical event in one category, the patient is counted only once per category.  **Consisted of, but not limited to, Anemia, Arrhythmia, Catheterization/Cardiac Surgery, and Hypotension | | | |
